# Comprehensive genetic screening of 70 severe adolescent idiopathic scoliosis probands reveals novel pathogenic variants and syndromic associations

**DOI:** 10.3389/fmed.2025.1646415

**Published:** 2025-10-24

**Authors:** Monika Horbacz, Magdalena Koczkowska, Marek Rocławski, Marcin Ceynowa, Piotr Madanecki, Daniil Sarkisyan, Jakub Mieczkowski, Karolina Śledzińska, Jan P. Dumanski, Rafał Pankowski, Arkadiusz Piotrowski

**Affiliations:** ^1^3P-Medicine Laboratory, Medical University of Gdańsk, Gdańsk, Poland; ^2^Department of Biology and Pharmaceutical Botany, Medical University of Gdańsk, Gdańsk, Poland; ^3^Department of Orthopedic Surgery, Medical University of Gdańsk, Gdańsk, Poland; ^4^Department of Immunology, Genetics and Pathology, Uppsala University, Uppsala, Sweden; ^5^Department of Paediatrics, Haematology and Oncology, Medical University of Gdańsk, Gdańsk, Poland

**Keywords:** idiopathic scoliosis, whole exome sequencing, candidate variants, SNP array, FLNB, ENAM

## Abstract

**Introduction:**

Idiopathic scoliosis (IS) is a complex spinal deformity affecting ~3% of the population, with a multifactorial and genetically heterogeneous origin. This study aimed to investigate the genetic origins of severe IS by examining both constitutional and post-zygotic alterations.

**Methods:**

We analyzed 70 unrelated IS-affected individuals using whole exome sequencing (WES) and SNP array approaches on intraoperatively collected articular processes and blood samples.

**Results:**

Two pathogenic constitutional copy number variants (CNVs) were identified – a 43.6 Mb duplication on chromosome 8p and trisomy X – along with eight regions of homozygosity (ROH) located on chromosomes 1, 2, 8, 12, 14, and 16, absent in ethnically matched controls. Additionally, a heterozygous *DMD* deletion (exons 17–36) was found in one female, and rare recurrent pathogenic single-nucleotide variants (SNVs) were detected in *ENAM* and *FLNB* genes. Notably, 13% (95% CI, 6.1–23%) of individuals harbored pathogenic variants, spanning CNVs, ROH, and SNVs, suggesting a genetic contribution to IS.

**Discussion:**

Our findings demonstrate that one in seven cases classified as idiopathic may have an underlying monogenic cause. This study underscores the polygenic and heterogeneous nature of IS and highlights the need for genetic testing by integrating WES and SNP array analyses into its diagnostic workflow. Our findings suggest that incorporating genetic testing into the diagnostic evaluation of severe IS patients may enable personalized genetic counseling and, consequently, improve clinical management.

## 1 Introduction

Scoliosis is a three-dimensional spine deformity, diagnosed through X-ray imaging by measuring the major curvature using the Cobb angle method (≥10°). Approximately 80% of cases are classified as idiopathic, meaning that no clear cause is identified (1, 2). Idiopathic scoliosis (IS) is primarily observed in adolescents (AIS), with girls being up to 10 times more likely to develop severe disease and experience rapid progression (3). The female predominance may reflect developmental factors, as girls enter puberty earlier during the critical period of postural system maturation. However, the absence of a clear sex-linked inheritance pattern suggests the Carter effect, a genetic phenomenon in which males, as the less frequently affected sex, must carry a higher genetic burden to develop the condition. This increases their likelihood of transmitting it to the next generation, thereby maintaining the sex imbalance (1, 4–6).

The prevalence of IS varies geographically and affects roughly 2–3% of the population. Research suggests that IS may have a hereditary component, with an estimated 6–11% penetrance among first-degree relatives, further supported by higher concordance in monozygotic compared to dizygotic twins (1). A Swedish Twin Registry-based study found that genetic factors account for 38% of the risk of developing scoliosis, while the remaining 62% attributed to environmental factors. Although a polygenic inheritance pattern has been predominantly proposed for IS, identifying its precise genetic basis remains challenging due to its heterogeneity. Several limitations, including small cohort sizes, inconsistent findings, difficulties in replicating results across different populations, and insufficient clinical examination of study groups, contribute to the existing gaps in knowledge.

In this study, we selected a well-defined cohort with a uniform ethnic background, focusing on severe, surgically treated IS cases, with a median curvature of 59 degrees. Since only a fraction of IS patients (0.1–0.3%) exhibit curvatures exceeding 40 degrees, we hypothesized that this severely affected group is enriched for constitutional and/or post-zygotic pathogenic variants compared to the general AIS population (2, 3, 7, 8).

Our study focused on a comprehensive molecular analysis of both constitutional and post-zygotic variants. To investigate this, we collected paired blood and intraoperative material from articular processes, enabling the assessment of single nucleotide variation using whole-exome sequencing (WES) and SNP array-based genotyping for structural variation. Post-zygotic mosaic variants arise after fertilization and may be present only in a subset of tissues, making them undetectable in blood alone. This strategy enabled exploring genetic mechanisms that have not previously been investigated in IS. The primary aim of the study was to identify constitutional and post-zygotic variants in severe AIS, providing a basis for future studies and potential applications in personalized genetic counseling and clinical management.

## 2 Materials and methods

### 2.1 Ethics statement

This study was approved by the Independent Bioethics Committee for Research at the Medical University of Gdańsk (no. NKBBN/418/2017). Written informed consent for genetic testing was obtained from all individuals participating in this study and/or their parents/legal guardians. Control samples used in the study were collected under a research protocol approved by the Bioethical Committee at the Collegium Medicum, Nicolaus Copernicus University in Toruń (no. KB509/2010) and by the Independent Bioethics Committee for Research at the Medical University of Gdańsk (no. NKBBN/564/2018), and all donors were recruited and enrolled under informed and written consent. All research involving human participants and human-derived tissues was conducted in accordance with the relevant guidelines and regulations, including Declaration of Helsinki. No personally identifiable information was included in the manuscript.

### 2.2 Clinical description of studied subjects

This study included 70 unrelated individuals of a uniform ethnic background, with an average age of 15. All probands were diagnosed with severe IS, characterized by a median Cobb angle deformation of 59°. They underwent direct vertebral rotation as part of scoliosis correction treatment (9). We specifically included young individuals whose severe scoliosis was not attributable to mechanical damage or environmental factors that could have contributed to the progression or manifestation of spinal curvature. Individuals with known genetic syndromes causing secondary scoliosis were excluded. However, genetic testing was not part of the standard diagnostic procedure offered before enrollment in the study.

**Figure 1:**
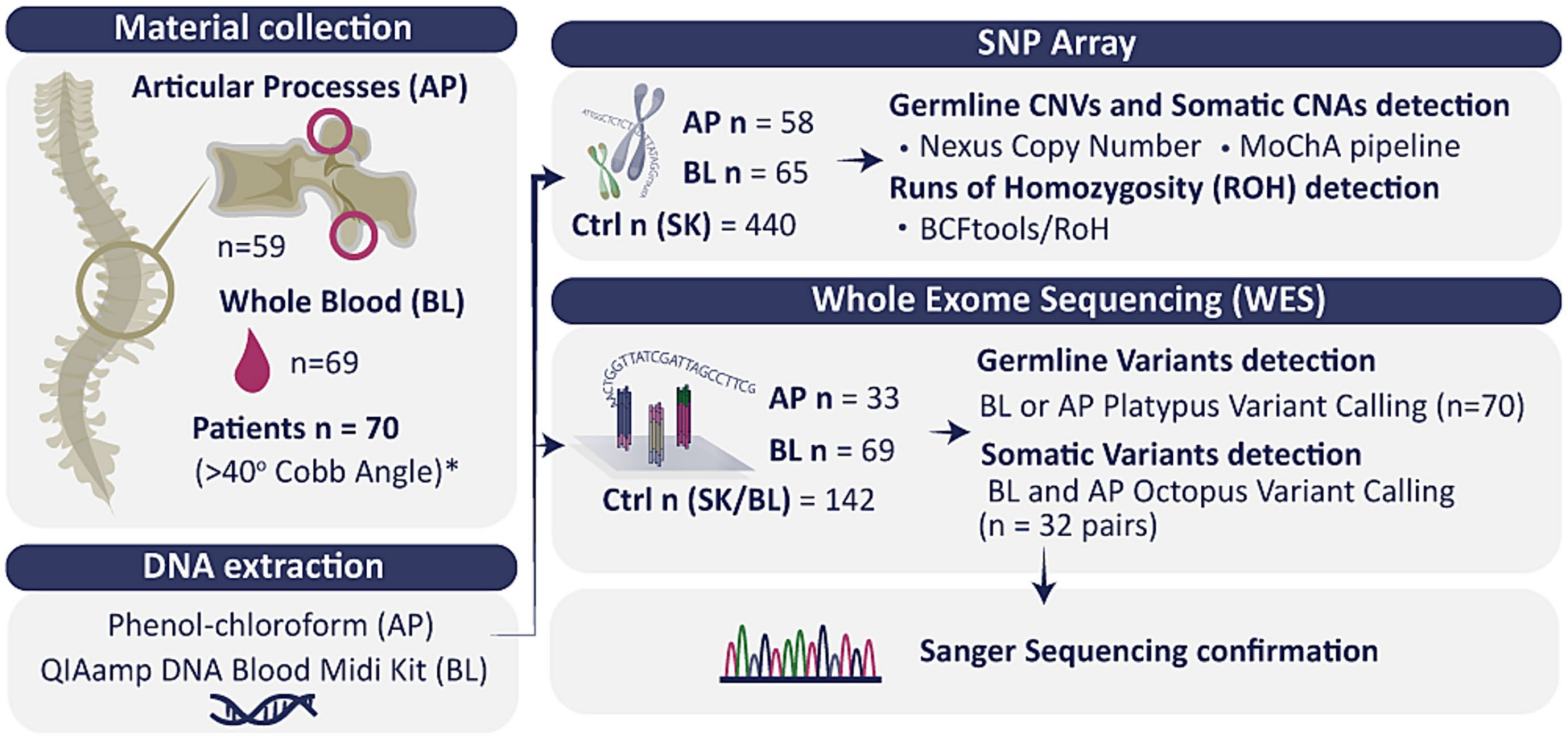
Visual representation of the study workflow. AP, articular processes; BL, whole blood; SK, skin; Ctrl, control; CNVs, copy number variations; CNAs, copy number alterations; ROH, runs of homozygosity, *or smaller if clinically justified surgery qualification.

Blood samples (BL) and/or articular processes (AP) closest to the area of deformation were collected from the individuals, depending on availability. Of the 70 individuals, both types of samples were collected from 58 individuals, while the remaining 12 provided only one type, either from BL or AP (Figure 1; Supplementary Table 1). To verify whether the candidate variant is *de novo* or inherited, BL samples were collected from the probands’ parents, when available.

Detailed clinical information of the studied cohort is provided in the Supplementary Table 1. Briefly, the cohort comprised 13 boys and 57 girls. Notably, 60.3% (41/68) of the cases did not report a family history of scoliosis. Family history was collected via self-report from patients or their parents (unavailable for two individuals), and no clinical examinations of relatives were performed. The cohort’s average height and weight percentiles were 61 and 58, respectively, based on reference growth and BMI charts for Polish adolescents (10). Besides severe scoliosis, several patients exhibited additional skeletal abnormalities, including pectus excavatum in two probands, knee problems, pigeon-toe, flat feet, Osgood-Schlatter disease, and incorrect pelvis rotation, each reported in one case. Furthermore, back pain was reported by 27.5% (19/69) of participants. Among these 19 individuals, some disclosed accompanying symptoms, including respiratory difficulties (2/19), paresthesia (1/19), myoclonus (1/19), and paraparesis (1/19). Clinical information was unavailable for one individual.

As a control for the study, BL and skin (SK) samples from two groups of female individuals diagnosed with breast cancer, with the same ethnic background, were analyzed using SNP array and WES following the same protocols as the studied group. The first control group, consisting of 440 unrelated individuals, provided SK samples for the structural genomic rearrangement analysis. The second control group, used for WES analysis, included 79 SK and 63 BL samples from 142 individuals. Neither group was preselected based on scoliosis status, with the population risk of mild scoliosis estimated at approximately 3% (2, 3, 8).

### 2.3 DNA isolation

DNA extraction from peripheral blood leukocytes was performed using the QIAamp DNA Blood Midi Kit (Qiagen, Germantown, MD), while the standard phenol-chloroform method was used for solid tissue samples. The quality and quantity of DNA were assessed spectrophotometrically (Varioscan-Thermo Fisher, Waltham, MA) and fluorometrically (Qubit-Thermo Fisher & TapeStation–Agilent Technologies, Santa Clara, CA).

### 2.4 SNP array

We conducted a chromosomal rearrangement analysis using the Illumina Infinium Global Screening Array Multiple Disease (GSA-MD-24v3-0-EA_20034606_A1) (Illumina, San Diego, CA) according to the manufacturer’s recommendations (11, 12) to identify DNA copy number variation (CNVs), copy number alterations (CNAs), and runs of homozygosity (ROH) within the genome (hg19). Genotyping was performed on 65 individuals, including 58 with both AP and BL and seven with only BL samples. Data were analyzed using Nexus Copy Number v10.0 (BioDiscovery), the MoChA pipeline v.2023–09-19 (13, 14), and Bcftools v.1.17 (15).

In the Nexus Copy Number analysis, only alterations supported by at least five probes in samples with Log Ratio (LRR) standard deviation <0.2 were included. Constitutional alterations over 150 kbp were included, while duplications and deletions <150 kbp were manually curated (Supplementary Table 2). CNVs entirely overlapping those present in the Database of Genomic Variants (as of November 2023) and lacking protein-coding genes were excluded (16). The pathogenicity of constitutional CNVs was further assessed using publicly available online tools, i.e., Franklin by Genoox and CNV-ClinViewer by Broad Institute, followed by the manual inspection. Both tools are semi-automated systems for the clinical significance classification of CNVs, aligning with the current diagnostic recommendations (17).

To analyze post-zygotic findings, data from each individual were examined in pairs of AP and BL, first using Nexus Copy Number and then the MoChA pipeline to identify CNAs occurring in low cell fractions, with default parameters (14).

For ROH confirmation, we applied the BCFtools/RoH command to the VCF generated by the MoChA pipeline (15). This command identifies autozygosity regions using a hidden Markov model and 1,000 Genomes allele frequencies as reference. Results with quality scores <90 (fwd-bwd phred) and <5 Mbp were excluded (18).

Results were visualized using R v4.1.2 and package *karyoploteR* (19).

### 2.5 Whole exome sequencing (WES)

WES libraries were prepared for all 70 enrolled probands using SureSelect XT HS All Exon V7 (Agilent Technologies) and Twist Library Preparation EF Kit 2.0 (Twist Bioscience, South San Francisco, CA), following manufacturers’ protocols. This included 32 pairs of AP and BL, and the remaining 38 samples being either BL or AP, depending on material availability. The libraries sequencing was performed using NextSeq550, HiSeq XTen, and NovaSeq 6,000 Illumina instruments, with a mean sequencing depth of 157x (median 137x) across targeted regions.

For data analysis, an in-house pipeline based on GATK4 best practices was applied (20). The reads were aligned to the reference genome (hg38) using the Burrows-Wheeler transform aligner (21). Complementary variant calling strategies were employed: Platypus v0.8.1.1 for constitutional variants, which demonstrates superior performance for constitutional variant detection in WES data, and Octopus v0.7.4 for post-zygotic variants, which uses a haplotype-aware algorithm analyzed in pairs—specifically optimized for low-frequency variant detection, including mosaicism (22, 23). Variants with mapping quality <30 or supported by high-quality bases (≥30), but in fewer than five reads, and variants located outside the targeted regions were excluded from further analysis. Functional annotation of all files was performed with ANNOVAR (24). Filtering was conducted using R v4.1.2.

### 2.6 Variant selection criteria

All events in exonic and splicing canonical regions that passed the variant calling filters, with a sequencing depth ≥30 and an allele frequency ≥0.07, were included. Constitutional variants classified as “Benign” and “Likely Benign” in the ClinVar database were excluded. All truncating variants were included for further pathogenicity assessment. Non-truncating events were filtered based on their population frequency using the Genome Aggregation Database (gnomAD v2.1.1, v3.1.2). Variants with a minor allele frequency (MAF) ≤ 0.01 (“popmax”) or absent in the database were retained.

To assess the theoretical deleterious impact of synonymous and missense variants, the Splice AI tool (*Δ* score ≥0.5) was used for splicing prediction, and REVEL (≥0.7) for analysis of missense variants (25, 26).

All post-zygotic variants called by Octopus were defined through comparison of paired AP and BL samples and underwent manual curation, including Integrative Genomics Viewer (IGV) inspection, exclusion of variants with gnomAD popmax MAF > 0.01, in silico pathogenicity prediction, and assessment of biological plausibility for scoliosis. These criteria were chosen to reduce false positives from artifacts or common polymorphisms while retaining rare, potentially disease-relevant events.

Firstly, we applied the above-mentioned filtering criteria to evaluate the pathogenicity of variants in genes previously linked to scoliosis (Supplementary Table 3) (27). Subsequently, using the same filtering criteria, we analyzed all remaining protein-coding genes to acquire a comprehensive understanding of the genetic makeup underlying IS. Variant pathogenicity classification was performed following the American College of Medical Genetics and Genomics (ACMG) and the Association for Molecular Pathology (AMP) diagnostic recommendations (28).

In this study, “recurrent variants” are defined as genetic variations that either arise independently in ≥2 unrelated individuals or represent ≥2 distinct variants within the same gene.

### 2.7 Genomic confirmation mutational analysis

Selected candidate variants classified as pathogenic, likely pathogenic, or variants of uncertain significance (VUS) were confirmed by bidirectional Sanger sequencing and analyzed using SnapGene v6.2.1.

### 2.8 Statistical analysis

Statistical tests were performed using R v4.1.2 with packages *stats* and *binom*. The statistical significance of the differences between the two groups was assessed using Fisher’s exact test with *p*-value <0.05. Confidence intervals for proportions were calculated using the exact binomial method (Clopper-Pearson) to ensure appropriate coverage for small sample sizes. Multiple testing correction was not applied, given the hypothesis-driven focus on recurrent variants.

## 3 Results

### 3.1 Structural chromosomal rearrangements

**Figure 2:**
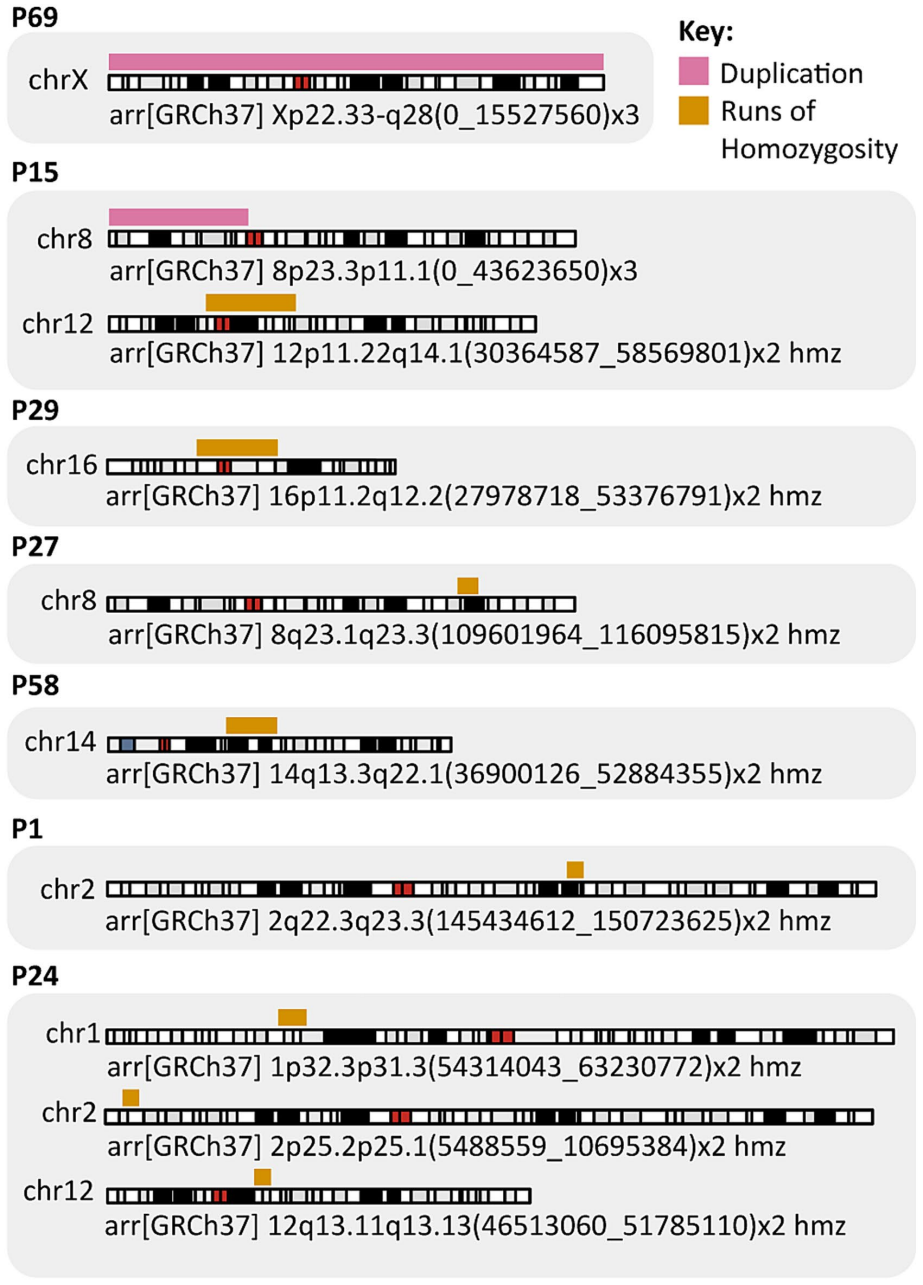
Key findings from the SNP array analysis.

We identified a total of 6,795 constitutional CNVs and 6,258 ROH using Nexus Copy Number software and BCFtools/RoH. After applying the filtering criteria (Materials and methods) we narrowed down the dataset to 41 CNVs and eight ROHs. Among the 41 reported here CNVs, three were classified as pathogenic or likely pathogenic and were not found in the control group. Namely, two large duplications were observed on chromosomes 8 and X in two unrelated probands, P15 and P69, respectively (Figure 2). A trisomy of the short arm of chromosome 8, spanning 43.6 Mbp, was identified in a 17-year-old girl with a severe IS characterized by left convex thoracic curvature, a Cobb angle of 60 degrees, mild facial dysmorphia, a wide neck, dextrocardia, and pectus excavatum. This individual also carried a 28 Mbp ROH on chromosome 12, including the centromeric region. A trisomy of chromosome X was identified in P69, a 14-year-old female with IS and a Cobb angle of 50 degrees, tall stature (73rd percentile), low body mass (below 3rd BMI percentile), aphasia, and back pain. Her medical history indicated that her father also had scoliosis, though no further clinical data were available. The third identified clearly pathogenic variant was a heterozygous deletion of exons 17–36 in the *DMD* gene in P46, a 12-year-old female with a Cobb angle of 60 degrees (Supplementary Table 2). All individuals with these findings were referred for genetic counseling.

Additionally, 19 CNVs were classified as likely benign or benign (data not presented), while another 19 CNVs were VUS. Eight ROHs >5 Mbp were detected on chromosomes 1, 2, 8, 12, 14, and 16 (Supplementary Table 2), encompassing a total of 712 protein-coding genes. Among them, 133 genes are potentially dosage sensitive.

No post-zygotic Copy Number Alterations (CNAs) in MoChA and Nexus analysis met the filtering criteria.

### 3.2 Single nucleotide variation analysis

No statistically significant post-zygotic variants, that met the filtering criteria (Methods), and were potentially damaging or related to scoliosis, were identified in the cohort of 32 AP.

After applying cut-off filtering criteria, constitutional variants identified in the studied cohort were grouped into two categories, as follows.

#### 3.2.1 Genes previously associated with IS

We identified here a total of 45 variants in 38 individuals, including 17 truncating variants found in 30% (21/70) of individuals and 28 non-truncating variants in 34.3% (24/70) of cases (Supplementary Table 4). Of these variants, 12 distinct and two recurrent heterozygous variants in 14 genes, present in 16 individuals, were classified as pathogenic or likely pathogenic according to current diagnostic recommendations (Table 1) (28). These included seven nonsense variants in the *DHCR7, FANCM, FLNB, NEB, OBSL1, RPGRIP1L,* and *WDR81* genes; three frameshifts variants in *FANCL, NALCN,* and *TMEM67;* two missense variants in *LMNA* and *SLC26A2;* and two splicing variants in *TMEM231* and *UPB1.* All 14 variants were either rare (“popmax” < 0.005) or had not been reported in the gnomAD database. Constitutional variants in the *FLNB, LMNA,* and *NALCN* genes have previously been associated with autosomal dominant (AD) disorders, such as Larsen syndrome, Laminopathies, Familial partial lipodystrophy, and Congenital contractures of the limbs, face, hypotonia, and developmental delay, which present clinically with scoliosis or other skeletal abnormalities (29–32). Among the genes specifically associated with IS, recurrent pathogenic, likely pathogenic, or VUS heterozygous variants were identified in *A2ML1*, *ERCC2, MYH7, FLNB, RYR1,* and *TMEM231* (Supplementary Table 4), with *FLNB* reaching statistical significance in Fisher’s exact test compared to the control group (*p*-value = 0.035). Statistical tests were performed based on the number of identified pathogenic variants in the studied group compared to the control group, counting occurrences per gene.

**Table 1:** Summary of constitutional likely pathogenic and pathogenic variants identified by whole exome sequencing (WES) in genes associated with idiopathic scoliosis.

Gene	Transcript	Nucleotide change	Protein change	ID	Zygosity	Inheritance	gnomAD AF	ClinVar	ACMG Class	Associated disorder
*DHCR7*	NM_001360.2	c.452G>A	p.(Trp151*)	P64	Het	Maternal	0.0014	30x P; 1x LP	P (PVS1; PP5; PP1)	Smith-Lemli-Opitz syndrome (AR)
*FANCL*	NM_018062.3	c.1096_1099dup	p.(Thr367Asnfs*13)	P41	Het	Maternal	0.0053	1x P; 9x VUS; 2x LB; 2x B	LP (PVS1; PM2)	Fanconi anemia (AR)
*FANCM*	NM_020937.2	c.1972C>T	p.(Arg658*)	P59	Het	Maternal	0.0001	3x P; 3x LP	P (PVS1; PM2; PP5)	Fanconi anemia (AR)
*FLNB*	NM_001457.4	c.3923_3924del	p.(Tyr1308*)	P13	Het	Paternal	Absent	No data	LP (PVS1; PM2)	Larsen syndrome (AD), Atelosteogenesis, type I or II (AD), spondylocarpotarsal synostosis syndrome (AR) and boomerang dysplasia (AD)
*LMNA*	NM_170707.4	c.688G>A	p.(Asp230Asn)	P64	Het	Unknown	Absent	1x P	LP (PM1; PM2; PP3; PP5)	Laminopathies (AD/ AR) which affect various tissues and organs, including muscles, fat, and bones
*NALCN*	NM_052867.4	c.1632del	p.(Phe544Leufs*16)	P46	Het	Maternal	Absent	No data	LP (PVS1; PM2)	Congenital contractures of the limbs and face, hypotonia, and developmental delay (AD)
*NEB*	NM_001164508.2	c.23989C>T	p.(Arg7997*)	P20	Het	Paternal	0.0005	6x P; 10x LP; 1x VUS	P (PVS1; PM2; PP5)	Nemaline myopathy (AR) and Arthrogryposis multiplex congenita 6 (AR)
*OBSL1*	NM_015311.3	c.4951G>T	p.(Glu1651*)	P16 P60	Het	Unknown	0.002	2x VUS; 2x LB	LP (PVS1; PM2)	Three M Syndrome 2 (AR)
*RPGRIP1L*	NM_015272.5	c.3594G>A	p.(Trp1198*)	P15	Het	Maternal	Absent	1x LP; 2x VUS	P (PVS1; PM2; PP5)	Joubert syndrome type 7 (AR), and Meckel Syndrome type 5 (AR)
*SLC26A2*	NM_000112.4	c.1957T>A	p.(Cys653Ser)	P30	Het	Unknown	0.0002	8x P; 2x LP	LP (PM1; PM2; PP3; PP5)	Diastrophic dysplasia, atelosteogenesis type 2 (AR), and Achondrogenesis type 1B (AR)
*TMEM231*	NM_001077418.3	c.664+1G>C	p.(?)	P5 P6	Het	2x (Paternal)	Absent	No data	LP (PVS1; PM2)	Joubert syndrome (AR) and Meckel syndrome (AR)
*TMEM67*	NM_153704.6	c.476_477del	p.(Ser159Phefs*15)	P40	Het	Paternal	Absent	No data	LP (PVS1; PM2)	Joubert syndrome (AR) or Meckel syndrome (AR)
*UPB1*	NM_016327.3	c.917-1G>A	p.(?)	P10	Het	Unknown	0.0027	12x P; 1x B	LP (PVS1; PM2)	β-ureidopropionase deficiency (AR)
*WDR81*	NM_001163809.2	c.3775G>T	p.(Gly1259*)	P1	Het	Unknown	Absent	No data	LP (PVS1; PM2)	Cerebellar ataxia, mental retardation, and disequilibrium syndrome (AR)

The variant pathogenicity classification was performed in line with the American College of Medical Genetics and Genomics (ACMG) and the Association for Molecular Pathology (AMP) diagnostic recommendations (28). Gene, Gene symbol according to HGNC nomenclature; Transcript, Reference transcript identifier from the MANE (Matched Annotation from NCBI and EMBL-EBI) project; Nucleotide change, DNA-level variant nomenclature according to HGVS guidelines; Protein change, Predicted amino acid change in the protein sequence; ID, Unique identifier for each proband carrying the variant; Zygosity, Heterozygous (Het) or homozygous (Hom) status; Inheriatance, Indicates the origin of the variant: maternal, paternal, *de novo*, or unknown. gnomAD AF, Allele frequency in the Genome Aggregation Database (gnomAD) version 2.1.1, using the maximum frequency observed across all populations (popmax; as of November 2023); ClinVar, Pathogenicity classification in the ClinVar database (as of October 2024): P, pathogenic, LP, likely pathogenic, VUS, variant of uncertain significance, LB, likely benign, B, benign; ACMG Class, Variant classification according to ACMG/AMP guidelines; Associated Disorder, Clinical disorder(s) associated with pathogenic variants in the gene, with inheritance patterns autosomal dominant (AD) / autosomal recessive (AR).

#### 3.2.2 Genes not previously linked to IS

In this group of genes, we identified 1,353 truncating variants, including 97 pathogenic or likely pathogenic variants, 554 VUS, and 702 benign or likely benign variants. Additionally, 516 non-truncating variants were identified, including 62 pathogenic or likely pathogenic variants, 355 VUS, and 99 benign or likely benign variants. These 159 pathogenic or likely pathogenic truncating and non-truncating variants (Supplementary Table 5) were identified in 151 genes in total. Of these, 20 genes contained 26 heterozygous variants, either recurrent or multiple distinct variants within the same gene, observed in 31 unrelated individuals.

## 4 Discussion

Here, we found that individuals with severe IS exhibit various structural and point variations, which lead to other known genetic disorders presenting with a scoliosis phenotype, albeit without a common genetic component. This observation aligns with the polygenic inheritance mode that has predominantly been proposed for IS (4).

### 4.1 Structural variants and gene dosage effects

Two large constitutional CNVs identified in our cohort highlight the role of chromosomal imbalance in severe IS (Figure 2; Supplementary Table 2). First, 47,XXX syndrome is a common chromosomal aneuploidy, affecting 1 in 1,000 females, often remaining undiagnosed due to subtle clinical symptoms. Affected individuals tend to be taller and may exhibit an increased prevalence of thoracic kyphosis (33). Trisomy X has been previously observed in AIS patients, with a reported frequency of 0.7% (2/286), compared to 0.19% (1/529) in controls (34). Our detection rate of 1.75% (1/57) (95% CI, 0–9.4%) and absence in controls substantially exceeds previous reports, suggesting enrichment in severe cases. The mechanism remains unclear whether scoliosis may result from direct genetic effects on spinal development or be secondary to the increased stature and altered growth patterns typical in 47,XXX syndrome.

The second large CNV, associated with a trisomy 8p syndrome, is characterized by a variable phenotype that includes mild to severe developmental delay, short stature, dysmorphic features, autism, epilepsy, scoliosis, and spastic paraplegia (35, 36). The trisomy 8p case exemplifies how gene dosage imbalances in critical developmental pathways contribute to severe IS. The duplication encompasses dosage-sensitive *FGFR1* and *BMP1* genes, both essential for spine and cartilage development, providing a plausible mechanism for the observed skeletal phenotype (Supplementary Table 1) (37, 38). The co-occurrence of a large ROH containing haploinsufficient connective tissue genes further illustrates the complex genetic architecture underlying severe IS, highlighting the complexity of genetic influences on spinal health.

In addition to the two large CNVs, we identified a small pathogenic deletion of exons 17–36 in the *DMD* gene in one female individual (Supplementary Table 1). Constitutional pathogenic variants in *DMD* lead to Duchenne or Becker muscular dystrophies, both inherited in an X-linked recessive manner. Female heterozygotes are usually asymptomatic; however, up to 17% exhibit muscle weakness (39). Papa et al. reported the presence of scoliosis and lordosis in 79% of female *DMD* heterozygous carriers, although the studied cohort was limited to 15 individuals (39). The co-occurrence of the *NALCN* pathogenic variant in this patient (Table 1), inherited from an affected mother, exemplifies potential oligogenic inheritance patterns in severe IS. Constitutional pathogenic variants in this gene cause autosomal dominant congenital limb and facial contractures, hypotonia, and developmental delay, with scoliosis being one of the main symptoms (32).

According to current diagnostic recommendations (18) ROH regions greater than 5 Mb are considered significant and may increase the risk of autosomal recessive (AR) disorders. The identification of eight large ROH regions absent in controls represents an unexplored mechanism in IS pathogenesis. These regions harbor numerous dosage-sensitive skeletal development genes (Supplementary Table 2), suggesting that regional haploinsufficiency or unmasking of recessive alleles may contribute to disease severity, particularly in populations with higher consanguinity rates.

### 4.2 Simple nucleotide variants and heterozygous carriers

The WES findings from our cohort align with prior studies, providing a list of potential candidate genes associated with IS (Table 1) (1, 2, 5). Among the identified variants, *FLNB* and *LMNA,* both associated with autosomal dominant disorders that include scoliosis in their phenotypic spectrum, emerge as particularly significant contributors to severe IS, with *FLNB* showing statistical enrichment compared to controls (*p* < 0.05).

*FLNB* encodes filamin B, a cytoplasmic protein that organizes the actin cytoskeleton, whose alterations cause skeletal disorders, including AD Larsen syndrome and AR spondylocarpotarsal synostosis. Jiang et al. demonstrated that *FLNB* pathogenic variants alter protein conformation in IS, suggesting a disease-modifier role (31). The identification of rare variants absent in controls supports a causal role in the pathogenesis of severe IS. However, due to limited evidence, two of three identified variants are classified as VUS until further data becomes available (Supplementary Table 4).

Similarly, *LMNA* encodes Lamin A and C, which are crucial for nuclear integrity and cellular processes. Variations in *LMNA* gene are linked to laminopathies, which affect muscles, fat, and bones, causing skeletal deformities, including scoliosis (29, 30), providing a mechanistic link between nuclear envelope dysfunction and spinal deformity.

Beyond AD genes, we identified several clearly pathogenic variants associated with AR disorders (Table 1). While heterozygotes for AR conditions are typically asymptomatic due to the presence of one functional allele, rare cases exhibit mild symptoms or increased susceptibility to conditions associated with the underlying disease. For instance, symptomatic heterozygotes have been reported in metabolic and neuromuscular disorders, such as cystinuria type 1 and 2 or hereditary aceruloplasminemia (40).

When comparing the prevalence of heterozygous pathogenic variants in the genes linked to AR diseases (Table 1), we observed a rate of 22.86% (16/70) (95% CI, 13.7–34.4%) in the IS cohort versus 14.08% (20/142) (95% CI, 8.8–20.9%) in controls. This difference was not statistically significant (Fisher exact test, *p*-value = 0.12), and therefore should be interpreted cautiously.

The underlying causes of symptomatic heterozygosity remain unclear. Hypothetical mechanisms could include oligogenic inheritance, synergistic heterozygosity, DNA methylation, or environmental factors. Other theoretical explanations might involve dosage effects leading to haploinsufficiency, dominant-negative effects, gain-of-function variations, or undetected secondary variants such as deep splice or regulatory changes (40–42). However, these possibilities are speculative, and further studies will be required to investigate their potential role in IS.

### 4.3 Preliminary novel findings

As a second tier of this study, we analyzed genes not previously linked to IS. While several variants classified as pathogenic or likely pathogenic (Supplementary Table 5) are primarily associated with metabolic, developmental, or immunological disorders, scoliosis is generally not considered a direct or secondary feature. One exception is the *ENAM* gene, which showed recurrent rare pathogenic frameshift variants in four unrelated individuals. The cumulative results for *ENAM* were statistically significant (*p* = 0.04, Fisher’s exact test) compared to the control group (Supplementary Figure 1).

Both frameshifts occur in the terminal exon of *ENAM* and are associated with amelogenesis imperfecta (AI), a condition affecting enamel formation, resulting in truncated enamelin protein production. *ENAM* c.1259_1260insAG has been extensively reported in the literature, whereas c.2763del has been described only once in compound heterozygosity with another *ENAM* variant, both linked to AR AI (43). The identified variants are rare in the gnomAD population database (“popmax” ~ 0.00036) and are listed as pathogenic in ClinVar and Leiden Open Variation Database (LOVD; as of November 2024).

However, as our questionnaire did not specifically query dental conditions, and no dental abnormalities were spontaneously reported by participants, we cannot determine whether AI features were present. Additionally, these individuals reported no family history of scoliosis, further limiting the interpretability of the *ENAM* association. While no direct link between *ENAM* and scoliosis exists, some studies suggest that dental issues and spinal deformities may co-occur (44). Fuchs et al. found that enamelin variations affected bone and energy metabolism in mutant mouse lines, suggesting a pleiotropic role of the *ENAM* (45). However, this remains speculative and highlights the need for replication in properly matched cohorts with comprehensive dental phenotyping.

### 4.4 Post-zygotic variation in severe IS

An important consideration for future research is the potential role of post-zygotic variation in severe IS. To our knowledge, this study is the first to systematically investigate post-zygotic variants in IS using paired blood and intraoperative spinal tissue samples. Analyses including SNP array and WES revealed no evidence of tissue-specific mosaic variants in this cohort, suggesting that post-zygotic mosaicism does not play a major role in severe IS. Our mean sequencing coverage of 157 × may not detect low-level mosaicism (<10% variant allele frequency), leaving open the possibility of rare or tissue-restricted events. These negative findings are valuable, narrowing the search for causative mechanisms and suggesting that constitutional variants, rather than post-zygotic events, drive disease pathogenesis. Future studies employing ultra-deep sequencing or single-cell approaches may still uncover rare mosaic events below our detection threshold.

### 4.5 Study limitations

While the study was rigorous, including a large, well-phenotyped cohort of young individuals from the same ethnic background with severe scoliosis, there are some limitations. The control group was not fully standardized: it consisted exclusively of females and was not screened for scoliosis, with an estimated population risk of mild scoliosis of approximately 3%, which may limit the comparability and generalizability of our findings (2). Additionally, family history was self-reported, and family members were not clinically examined, which may affect the precision of clinical interpretations. These factors should be taken into account when extrapolating our results to broader populations.

### 4.6 Clinical implications and future directions

Despite the lack of direct recommendations for routine genetic testing in IS (2), our results suggest that molecular analysis could be valuable for individuals with severe IS (Cobb >40°) (2), as severe curvature suggests a strong genetic basis. Our findings suggest that genetic testing may have clinical utility for patients with severe IS. Diagnostic yield of 13% (9/70 individuals) (95% CI: 6.1–23%) comprising pathogenic CNVs including large chromosomal abnormalities and gene deletions (4.3%; 3/70) (95% CI: 0.9–12%), pathogenic variants in AD IS genes (2.9%; 2/70) (95% CI: 0.3–9.9%), and ROHs potentially contributing to disease susceptibility (5.7%; 4/70) (95% CI: 1.6–14%) partially supports our hypothesis that severely affected individuals are enriched for constitutional pathogenic variants compared to the general AIS population. This 13% yield is specific to severe IS cases and should not be generalized to the broader IS population with milder phenotypes. However, contrary to our expectations, no post-zygotic variants were identified, and the marked genetic heterogeneity observed challenges the concept of a unified pathogenic mechanism. Therefore, rather than targeted gene panels, a comprehensive diagnostic strategy is warranted. A stepwise approach could be considered, beginning with SNP array analysis as a cost-effective screening tool, given that large structural variants account for a notable proportion of identifiable genetic causes. If results from microarray analysis are negative, WES could provide more comprehensive information, as it is not limited to a predefined set of genes, which – as shown in the literature (46) – tend not to be recurrent. This approach may be particularly relevant in diverse populations, where population-specific gene panels may not reliably capture all pathogenic variants. Early identification of genetic factors may help guide treatment strategies and provide valuable information for genetic counseling.

## Data Availability

The datasets presented in this study can be found in online repositories. The names of the repository/repositories and accession number(s) can be found at: https://www.ebi.ac.uk/ena, EGAS00001008152.
