# Supplementary material for: Comprehensive genetic screening of 70 severe adolescent idiopathic scoliosis probands reveals novel pathogenic variants and syndromic associations
Source: Front Med (Lausanne). 2025 Oct 24;12:1646415. doi: 10.3389/fmed.2025.1646415 (PMC12592074; doi:10.3389/fmed.2025.1646415)
Supplement: Supplementary file 6 [file Table_6.DOCX]

**Supplementary Information**

**Comprehensive Genetic Screening of 70 Severe Adolescent Idiopathic Scoliosis Probands Reveals Novel Pathogenic Variants and Syndromic Associations**

Monika Horbacz^1,2†^, Magdalena Koczkowska^1†^, Marek Rocławski^3^, Marcin Ceynowa^3^, Piotr Madanecki^2^, Daniil Sarkisyan^4^, Jakub Mieczkowski^1^, Karolina Śledzińska^5^, Jan P. Dumanski^1,4^, Rafał Pankowski^3*^, Arkadiusz Piotrowski^1*^

^1^ 3P-Medicine Laboratory, Medical University of Gdansk, Gdansk, Poland.

^2^ Department of Biology and Pharmaceutical Botany, Medical University of Gdansk, Gdansk, Poland.

^3^ Department of Orthopedic Surgery, Medical University of Gdansk, Gdansk, Poland.

^4^ Department of Immunology, Genetics and Pathology, Uppsala University, Uppsala, Sweden.

^5^ Department of Paediatrics, Haematology and Oncology, Medical University of Gdansk, Gdansk, Poland.

†These authors contributed equally to this work and share first authorship

*These authors contributed equally to this work and share senior authorship

***Correspondence:**

Rafał Pankowski: rafal.pankowski@gumed.edu.pl

Arkadiusz Piotrowski: arkadiusz.piotrowski@gumed.edu.pl

**Keywords:** Idiopathic scoliosis, whole exome sequencing, candidate variants, SNP array, *FLNB*, *ENAM*

**Note:** Supplementary Tables 1 - 5 are provided as separate Excel files due to their large size.

**Supplementary Table 1:** Individual characteristics and clinical data, including patient ID, type of sample collected (blood [BL], articular processes [AP]), availability of WES and SNP Array data, Cobb angle (scoliosis severity), age, gender, BMI percentile, family history, comorbidities, skeletal defects, and treatment details; NA- not applicable.

**Supplementary Table 2:** Summary of Copy Number Variations (CNVs) and regions of homozygosity (ROH) from SNP array analysis, including chromosome position, size, type (ROH, duplication-DUP, deletion-DEL), cytoband, classification (Franklin, ClinViewer), associated syndromes/diseases, and scoliosis-related genes. Identified variants are linked to patient IDs and genomic context; NA- not applicable.

**Supplementary Table 3:** List of IS-associated genes based on the literature, including HGNC gene symbols, full gene names, and OMIM database IDs.

**Supplementary Table 4:** Summary of constitutional WES results classified as (likely - LP) pathogenic (P) and VUS, including amino acid changes (MANE), patient IDs, control presence, ACMG classification and details, exonic function, Splice AI and REVEL scores, population frequency (gnomAD), variant reports (LOVD, ClinVar), and comments on phenotype or literature; NA- not applicable.

**Supplementary Table 5**: Summary of constitutional WES results classified as likely pathogenic (LP) or pathogenic (P) according to ACMG, in genes previously unrelated to scoliosis. Includes amino acid changes (MANE), patient IDs, control presence, ACMG classification/details, exonic function, Splice AI/REVEL scores, gnomAD frequency, variant reports (LOVD, ClinVar).

**Supplementary Figure 1: *ENAM* protein structure and frameshift variant analysis in severe IS.**

**
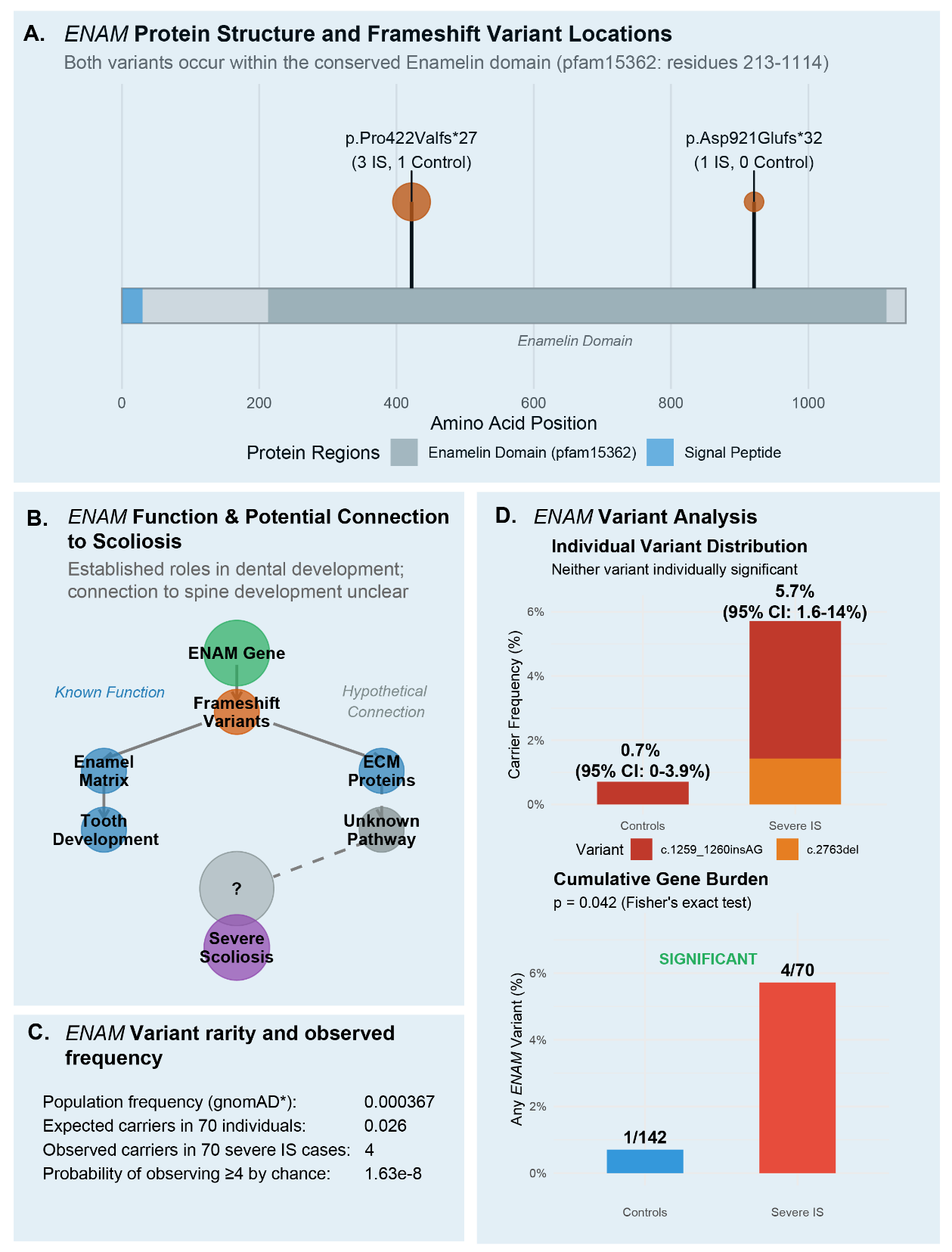
**

**A.** Panel illustrates the domain architecture of the *ENAM* protein, showing the full-length 1114 amino acid sequence represented by the gray horizontal bar with amino acid positions indicated on the x-axis. The N-terminal signal peptide is depicted in blue, while the conserved Enamelin domain (pfam15362, encompassing residues 213-1114) is shown in dark gray. Two frameshift variants identified in this study are marked with orange circles and vertical black lines indicating their precise locations. *ENAM* reference transcript NM_031889.3, IS – idiopathic scoliosis; **B.** Panel presents a schematic representation of the *ENAM* gene function and its potential connection to scoliosis pathogenesis. Established functional relationships are depicted with solid arrows and blue text, connecting *ENAM* to its known roles in enamel matrix formation and tooth development. Hypothetical connections are illustrated with dashed lines and gray elements, suggesting potential involvement in extracellular matrix (ECM) protein networks and an unknown pathway that may contribute to severe scoliosis development, represented by the purple circle; **C.** Panel provides statistical analysis of *ENAM* variant rarity and observed frequency. The panel displays key population genetics data, including the gnomAD population frequency of 0.000367, the expected number of carriers (0.026) in a cohort of 70 individuals, and the observed carriers (4) in 70 severe IS cases. The calculated probability of observing 4 or more carriers by chance (1.63×10⁻⁸) demonstrates the extreme statistical significance of this enrichment, indicating that the association between *ENAM* variants and severe scoliosis is highly unlikely to be due to random chance; **D.** Panel presents comprehensive variant analysis through two complementary visualizations. The upper graph shows individual variant distribution with carrier frequency (%) on the y-axis and sample groups on the x-axis. Controls exhibit a 0.7% total carrier frequency (1/142 individuals), while severe IS cases show a 5.7% carrier frequency. The variants c.1259_1260insAG and c.2763del are color-coded in dark red and orange, respectively, with neither variant reaching individual statistical significance. The lower graph displays cumulative gene burden analysis, comparing any *ENAM* variant frequency between controls (1/142, approximately 0.7%) and severe IS cases (4/70, 5.7%). Fisher's exact test yielded p = 0.042, establishing statistical significance as indicated by green text. This cumulative analysis demonstrates that while individual variants may not reach significance due to their rarity, the collective burden of *ENAM* frameshift variants is significantly enriched in severe idiopathic scoliosis cases compared to controls.
